# New insights into the roles of cucumber TIR1 homologs and miR393 in regulating fruit/seed set development and leaf morphogenesis

**DOI:** 10.1186/s12870-017-1075-6

**Published:** 2017-07-26

**Authors:** Jian Xu, Ji Li, Li Cui, Ting Zhang, Zhe Wu, Pin-Yu Zhu, Yong-Jiao Meng, Kai-Jing Zhang, Xia-Qing Yu, Qun-Feng Lou, Jin-Feng Chen

**Affiliations:** State Key Laboratory of Crop Genetics and Germplasm Enhancement, Nanjing Agricultural University, Nanjing, 210095 China

**Keywords:** Auxin receptor, Cucumber, *CsTIR1* and *CsAFB2*, miR393/TIR1 module, Fruit/seed set

## Abstract

**Background:**

TIR1-like proteins act as auxin receptors and play essential roles in auxin-mediated plant development processes. The number of auxin receptor family members varies among species. While the functions of auxin receptor genes have been widely studied in *Arabidopsis*, the distinct functions of cucumber (*Cucumis sativus* L.) auxin receptors remains poorly understood. To further our understanding of their potential role in cucumber development, two *TIR1-like* genes were identified and designated *CsTIR1* and *CsAFB2*. In the present study, tomato (*Sonanum lycopersicum*) was used as a model to investigate the phenotypic and molecular changes associated with the overexpression of *CsTIR1* and *CsAFB2*.

**Results:**

Differences in the subcellular localizations of *CsTIR1* and *CsAFB2* were identified and both genes were actively expressed in leaf, female flower and young fruit tissues of cucumber. Moreover, *CsTIR1*- and *CsAFB2*-overexpressing lines exhibited pleotropic phenotypes ranging from leaf abnormalities to seed germination and parthenocarpic fruit compared with the wild-type plants. To further elucidate the regulation of *CsTIR1* and *CsAFB2,* the role of the miR393/TIR1 module in regulating cucumber fruit set were investigated. Activation of miR393-mediated mRNA cleavage of *CsTIR1* and *CsAFB2* was revealed by qPCR and semi-qPCR, which highlighted the critical role of the miR393/TIR1 module in mediating fruit set development in cucumber.

**Conclusion:**

Our results provide new insights into the involvement of *CsTIR1* and *CsAFB2* in regulating various phenotype alterations, and suggest that post-transcriptional regulation of *CsTIR1* and *CsAFB2* mediated by miR393 is essential for cucumber fruit set initiation. Collectively, these results further clarify the roles of cucumber TIR1 homologs and *miR393* in regulating fruit/seed set development and leaf morphogenesis.

**Electronic supplementary material:**

The online version of this article (doi:10.1186/s12870-017-1075-6) contains supplementary material, which is available to authorized users.

## Background

Since the identification of F-box proteins TIR1/AFB (transport inhibitor resistant1/auxin signaling F-box) as auxin receptors [1, 2], a SCF^TIR1/AFB^-Aux/IAA-ARF signaling module has been well established, which sheds light on the linkage between auxin perception and gene expression [3]. In the absence of auxin, or at low concentrations, ARFs combine with Aux/IAA to form heterodimers; hence, transcription of auxin-responsive genes is not promoted until ARFs are released due to degradation of Aux/IAA by SCF^TIR1/AFB^ –ubiquitin mediated degradation induced by the presence of high auxin levels [4–6]. Thus, auxins act as a “molecular glue” to stimulate the interaction between TIR1/AFB and Aux/IAA [7–9].

As essential regulators of auxin responses in plants, TIR1-like proteins have been identified in various species and are divided into four distinct phylogenetic clades TIR1, AFB2 (AFB2/AFB3), AFB4 (AFB4/AFB5) and AFB6. TIR1-like proteins are involved in multiple auxin-responsive biological processes. In *Arabidopsis*, the TIR1/AFB auxin receptor family comprises six members: TIR1 and five additional AFB proteins [1, 3, 10]. The *tir1 afb* mutants of *Arabidopsis* exhibit defects in hypocotyl elongation, apical hook, and lateral root formation, leaf morphology and inflorescence architecture [11]. TIR1 and AFB2 act as positive regulators of auxin signaling by mediating auxin-dependent degradation of Aux/IAAs [12, 13]; While, the AFB4 and AFB5 are known to be the major targets of the synthetic auxin, picloram [14, 15], the in vivo roles of AFB1 and AFB3 are still unclear [12].

A similar role for TIR1 in leaf morphogenesis has also been elucidated in tomato and rice plants. Overexpression of *SlTIR1* in tomato plants resulted in altered leaf morphology [16], while suppression of *OsTIR1* increased the flag leaf inclination angle [17]. The role of TIR1 in fruit and seed development has also analyzed. Phenotypic and molecular analyses indicate that TIR1-like proteins are pivotal regulators of auxins in the fruit set process [16, 18, 19]. Further studies revealed that *SlTIR1* stimulates stenospermocarpic fruit formation in tomato plants [20]. Although the observation of diverse of degradation behaviors among TIR1/AFB-Aux/IAA complexes suggests that the existence of divergent properties among the *TIR1/AFB* genes [13], independent evidence has demonstrated functional redundancy among *TIR1/AFB* family genes. Loss-of-function analysis by generating higher order mutants in *Arabidopsis* confirmed that the TIR1/AFB proteins act redundantly to regulate diverse aspects of plant growth and development [11]. Individual knockdown of *OsTIR1* or *OsAFB2* in rice induced similar leaf morphology at the booting stage [17]. Thus, the mechanisms by which the small family of functionally redundant TIR1-like proteins mediate pleiotropic regulation processes to promote various auxin responses remain to be eucidated.

As the first genome-sequenced vegetable crop, cucumber now serves as a model organism for investigation of the *Cucurbitaceae* family. Compared with plants belonging to the *Cruciferae* and *Solanaceae* families, cucumber has distinct auxin-related developmental processes, such as determinate/indeterminate growth, tendril development and parthenocarpic fruit formation. However, few studies of the TIR1-like gene family of *Cucurbitaceae* have been reported to date*.* To gain insights into the roles of cucumber auxin receptors in mediating diverse developmental processes, *CsTIR1* and *CsAFB2* were cloned and functionally characterized in transgenic tomato plants. In accordance with the concepts of Dharmasiri et al. (2005) and Bian et al. (2012) [11, 17], the functions of *CsTIR1* and *CsAFB2* were revealed in this study. Further studies indicated that miR393-mediated post-transcriptional regulation of *CsTIR1* and *CsAFB2* contributes to the fruit set and development processes in cucumber.

## Results

### Phylogenetic and polymorphism analysis of *CsTIR1/AFB2*

**Fig. 1:**
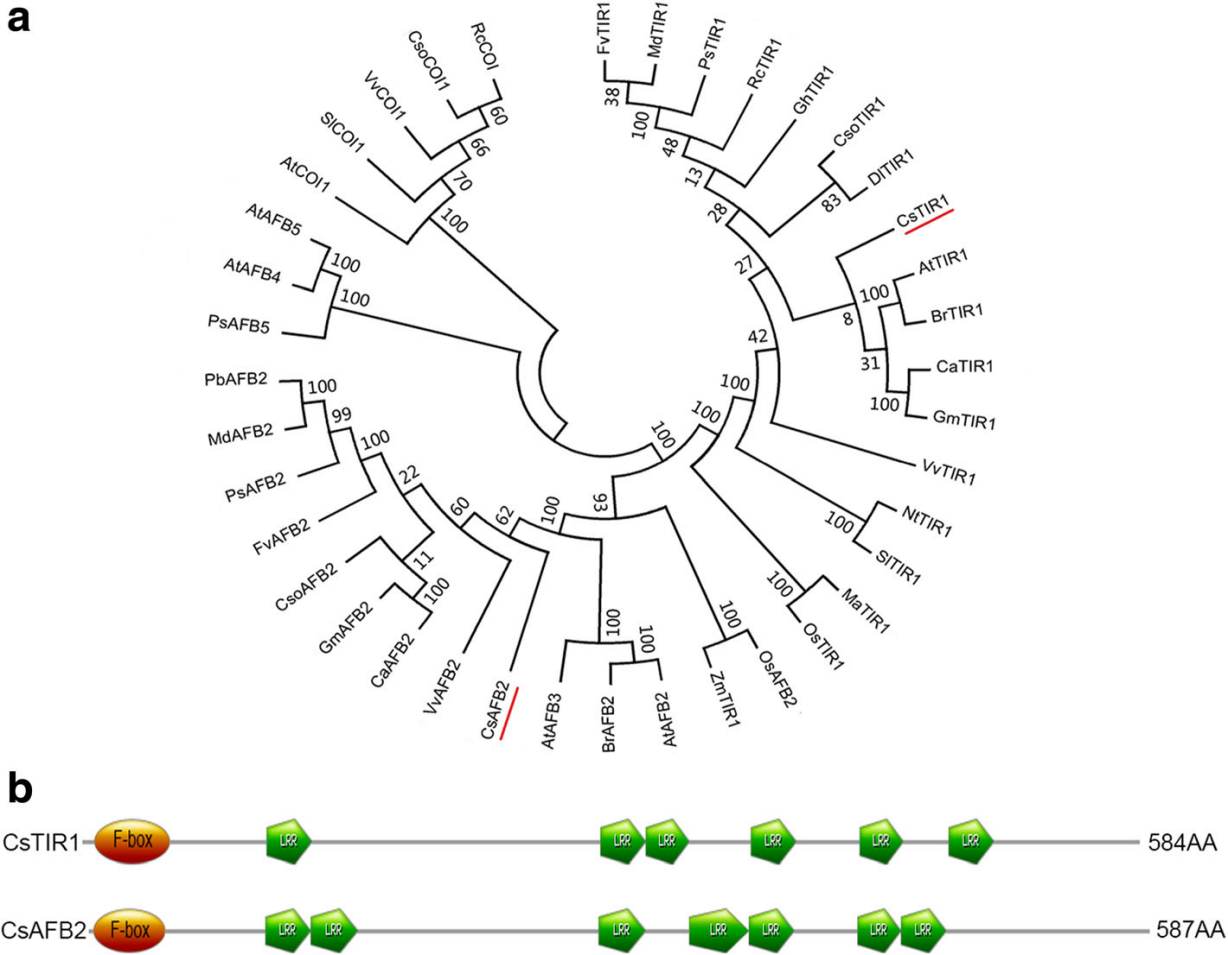
Evolutionary relationships among the TIR1 protein family members and domain structure of *CsTIR1/AFB* proteins. **a** The phylogenetic tree consists of 40 protein sequences of TIR1-like auxin receptors from various land plants and was generated using the neighbour-joining method in MEGA5. Auxin receptors isolated from cucumber are labeled with a red line. **b** CsTIR1 protein contains an F-box region and six leucine-rich repeat domains, CsAFB2 protein contains an F-box region and seven leucine-rich repeat domains. The numbers on the right indicate the number of amino acid residues

Two genes encoding proteins closely related to the *TIR1*-like gene family of auxin receptors were isolated from cucumber (GenBank ID: GX901282 and GX901283). To investigate their evolutionary relationships with well-defined auxin receptor family proteins of other plant species, a phylogenetic tree was generated using the neighbor-joining approach by MEGA 4.0. Phylogenetic analysis indicated that GX901282 is clustered to the TIR1 clade and has 76.6% similarity to *AtTIR1*, while GX901283 belongs to the AFB2 clade and has 75.8% similarity to *VvAFB2* (Fig. 1a; Additional file 1). Thus, the isolated sequences were designated *CsTIR1* and *CsAFB2* to be consistent with the nomenclature used for the homologs from other plant species. *CsTIR1,* which encodes a protein of 584 amino acid residues, contains an F-box region and six leucine-rich repeat (LRR) domains, while *CsAFB2* encodes a protein of 587 amino acid residues and comprises an F-box region and seven LRR domains (Fig. 1b).

### Subcellular localizations of CsTIR1/AFB2 proteins

**Fig. 2:**
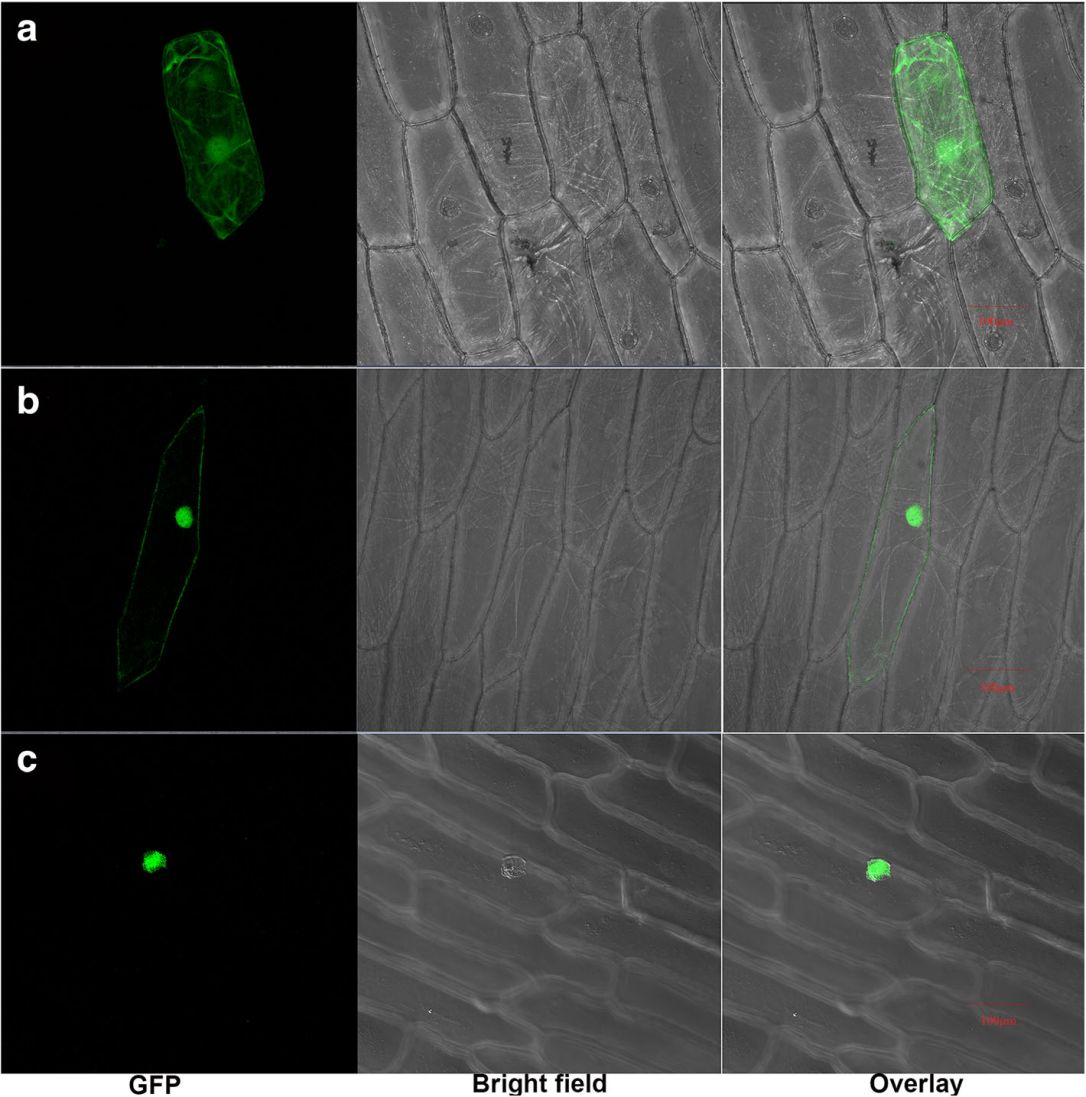
Subcellular localization of CsTIR1 and CsAFB2 in onion epidermal cells. Control plasmid (*GFP*) and fusion vector constructs (*CsTIR1-GFP*, *CsAFB2-GFP*) were transformed separately into onion epidermal cells by microprojectile bombardment. **a** Subcellular localization of GFP alone. **b** Subcellular localization of the CsTIR1-GFP fusion protein. **c** Subcellular localization of the CsAFB2-GFP fusion protein. All proteins were analyzed by laser scanning confocal fluorescence microscopy (GFP). Light micrographs (Bright field) and fluorescence (GFP) images are merged (Overlay) to illustrate the different location of the three proteins. Scale bars = 100 μm

To determine the subcellular localization of CsTIR1 and CsAFB2 proteins, the CsTIR1-GFP and CsAFB2-GFP fusion proteins were transiently expressed in onion epidermal cells using gene gun bombardment. Laser confocal scanning of protein fluorescence revealed that the green fluorescence signal of GFP alone was detected throughout the cell (Fig. 2a), in accordance with the expected cytosolic localization of the GFP protein. Interestingly, the fluorescence of CsTIR1-GFP was not only detected in nucleus but also detected in cytolemma (Fig. 2b), that is inconsistent with Ren’s result [16]. The green fluorescence signal of CsAFB2-GFP was detected in the nucleus (Fig. 2c).

### Expression patterns of *CsTIR1/AFB2* in cucumber

**Fig. 3:**
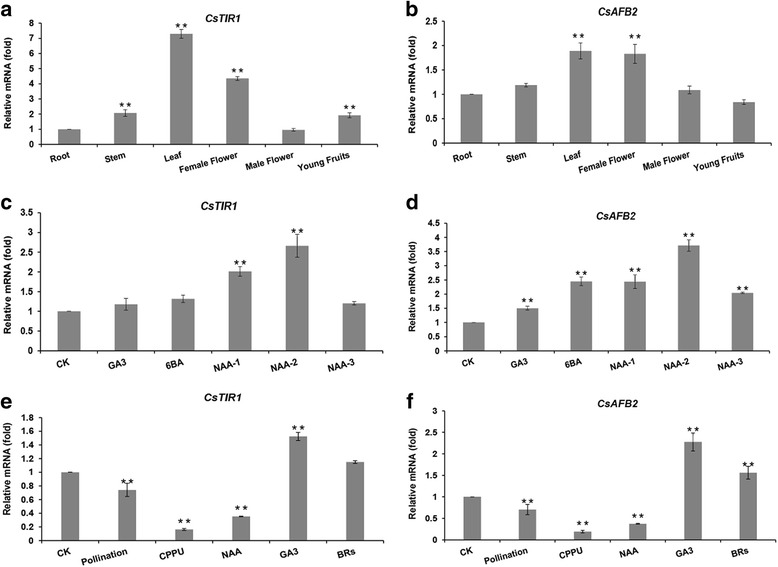
Quantitative RT-PCR analysis of *CsTIR1* and *CsAFB2* expression in cucumber. **a, b** *CsTIR1* and *CsAFB2* expression in different organs of cucumber (roots, stems, leaves, female flowers, male flowers, and young fruit). **c, d** *CsTIR1* and *CsAFB2* expression in cucumber leaves treated with GA3 (10 μM), 6BA (10 μM), and different concentrations of NAA (5, 10, and 50 μM). **e, f** *CsTIR1* and *CsAFB2* expression in cucumber ovaries under pollination and treatment with CPPU (400 μM), NAA (500 μM), GA3 (3000 μM), and BRs (0.2 μM). The “young fruit” represent fruit at 0 days post-anthesis. CK, “Control”. Data represent mean ± SD normalized relative to *CsActin* gene transcript levels. Root and CK expression data normalized to 1. All samples were analyzed in triplicate. Expression differences are calculated relative to expression in the root (**a**, **b**) and CK (**c**, **d**, **e**, **f**). (Student’s *t* test; * *P* < 0.05; ** *P* < 0.01)

Temporal and spatial transcriptional characteristics of *CsTIR1* and *CsAFB2* were investigated by qRT-PCR. Both *CsTIR1* and *CsAFB2* were detected in all the major organs of cucumber plants including root, stem, leaf, female/male flowers and young fruit. These two genes showed similar expression patterns, with the highest abundance in leaf and female flower tissues and relatively low mRNA levels in roots and young fruit (Fig. 3a, b). The phytohormones responses of *CsTIR1* and *CsAFB2* were investigated in exogenous hormone treatment experiments. qRT-PCR analysis showed that the transcription of *CsTIR1* was sensitive only to low (5 μM NAA) and medium (10 μM NAA) auxin concentrations (Fig. 3c). In contrast, *CsAFB2* expression was upregulated by exogenous gibberellins, cytokinin and auxin (Fig. 3d).

Treatment of cucumber ovaries (0dpa) with high concentrations of exogenous auxin (500 μM), cytokinins (400 μM), gibberellins (3000 μM) and brassinosteriod (0.2 μM) stimulated parthenocarpy. Interestingly, analysis of hormone-treated ovaries showed that expression of *CsTIR1* and *CsAFB2* was downregulated in the auxin- and cytokinins- induced parthenocarpic fruit as well as in the fruit set by pollination, while expression of the two genes was upregulated in gibberellin- and brassinosteriod- induced parthenocarpic fruit (Fig. 3e, f).

### Functional analysis of *CsTIR1* and *AFB2* genes

**Fig. 4:**
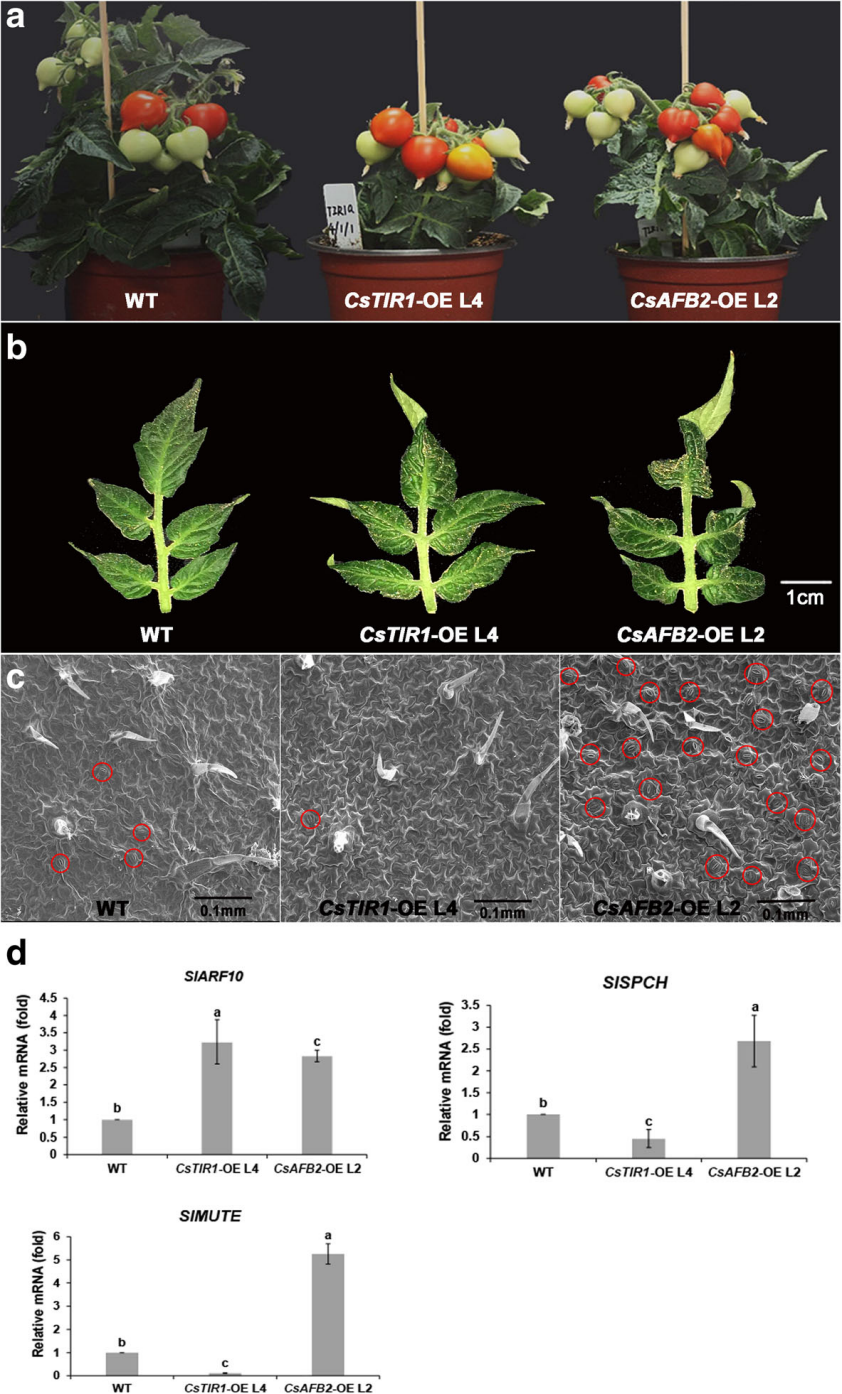
Vegetative growth and leaf architecture phenotypes in wild-type and transgenic plants. **a** Both *CsTIR1*-OE L4 and *CsAFB2*-OE L2 exhibited compact stature, and *CsTIR1*-OE L4 exhibited a severely dwarfed phenotype compared with the wild-type (WT). **b** Leaves of both *CsTIR1*-OE L4 and *CsAFB2*-OE L2 exhibited distorted growth behavior. Scale bar = 1 cm. **c** Leaf surface of WT, *CsTIR1*-OE L4, and *CsAFB2*-OE L2 observed by scanning electron microscopy. Transgenic line leaf surfaces were convex compared with the WT. Stomata are labeled with red circles. Scale bar = 0.1 mm. **d** Quantitative PCR analysis of transcript accumulation of *SlARF10*, *SlSPCH*, and *SlMUTE*. Expression of *SlARF10*, *SlSPCH*, and *SlMUTE* in the WT was normalized to 1. Data represent mean ± SD of three biological replicates. Different letters above bars indicate significant differences among different genotypes (Student’s *t*-test, *P* < 0.05)

To assess the physiological importance of the cucumber auxin receptor proteins, homozygous transgenic *Solanum lycopersicum,* cv. Micro-Tom lines overexpressing *CsTIR1* and *CsAFB2* were generated (designated *CsTIR1-*OE and *CsAFB2-*OE, respectively). qRT-PCR analysis showed that *CsTIR1* and *CsAFB2* were expressed abundantly in transgenic lines, with almost no effect on endogenous *SlTIR1* expression (Additional file 2). For each of the two genotypes, one of the most highly upregulated transgenic lines was selected for further characterization. Pleiotropic phenotypes were observed in these tomato lines (Table 1). Compact stature was the most intuitionistic alteration in the transgenic plants (Fig. 4a), which was consistent with the effects of transgenic expression of *SlTIR1* and *PslTIR1* [16, 19]. Compared with *CsAFB2*-OE lines, *CsTIR1-*OE lines exhibited reduced plant height phenotype (Fig. 4a). Distorted leaf growth resulted in severely inward curling growth status in transgenic lines (Fig. 4a, b).

**Table 1:** Summary of phenotypes in transgenic tomato plants overexpressing *CsTIR1* and *CsAFB2*

Genotype	Line number	Phenotype
35S–*CsTIR1*	L1-L4	Severely dwarf plants, abnormal leaves, suppressed seed size and germination activity, splited staminal cone, precocious fruit set prior to anthesis, reduced seed number/per fruit, parthenocarpic fruit set
35S–*CsAFB2*	L1-L2	Compact stature of plants, abnomal leaves, suppressed seed size and germination activity, splited staminal cone, precocious fruit set prior to anthesis, altered fruit shape, reduced seed number/per fruit, parthenocarpic fruit set

Both the *CsTIR1* and the *CsAFB2* overexpression lines exhibited a convex leaf surface phenotype, compared with the smooth leaf surface of wild-type (WT) plants. Although overexpression of *SlTIR1* has been reported to increase trichrome numbers [16], no changes in the morphology and number of trichomes were observed in the leaves of *CsTIR1*-OE and *CsAFB2*-OE transgenic tomato plants. Surprisingly, *CsTIR1* overexpression reduced stomata formation, while the number was significantly reduced by *CsAFB2* overexpression (Fig. 4c). Expression analysis of related genes strongly indicated that the ARF10 protein functions as a transcriptional repressor of leaflet lamina outgrowth [21], while SPCH and MUTE were found to be involved in the stomata differentiation process [22, 23]. qRT-PCR analysis showed that *SlARF10* was upregulated in transgenic lines and was implicated as a positive regulator of leaf morphology. Furthermore, *SlSPCH* and *SlMUTE* were upregulated in *CsAFB2-*OE lines but down-regulated in *CsTIR1-*OE lines (Fig. 4d).

**Fig. 5:**
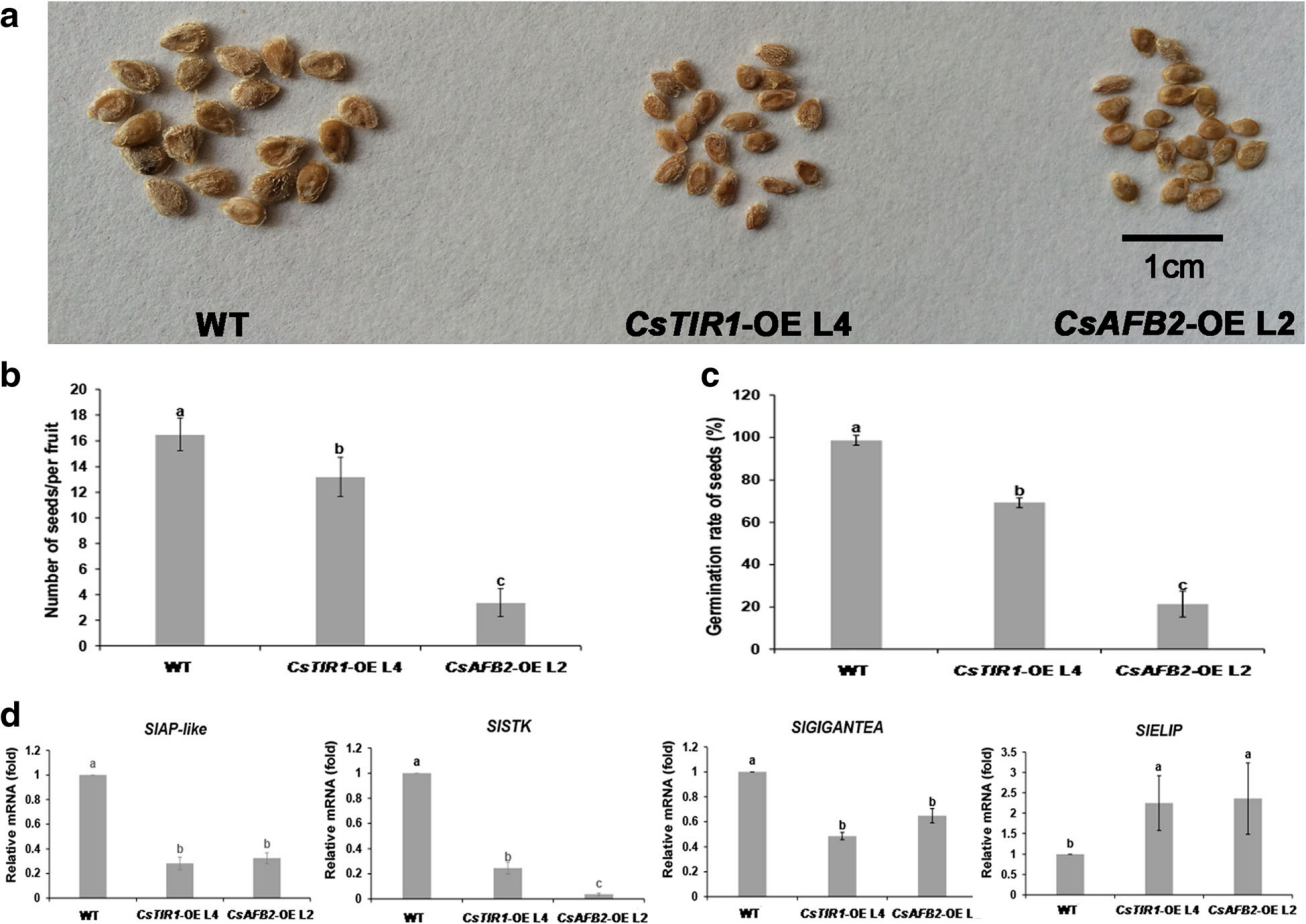
Seed size, seed number and seed germination activity were changed in transgenic plants. **a** Seed size of transgenic plants was smaller than that of wild-type. Scale bar = 1 cm. **b** Seeds in each fruit of transgenic plants were fewer than the wild-type plants. **c** Seed germination potential was reduced in transgenic lines. **d** Quantitative PCR analysis of transcript accumulation of *SlAP-like*, *SlSTK*, *SlGIGANTEA* and *SlELIP*. Expression of *SlAP-like*, *SlSTK*, *SlGIGANTEA* and *SlELIP* in the WT was normalized to 1. Data represent mean ± SD of three biological replicates. Different letters above bars indicate significant differences among different genotypes (Student’s *t*-test, *P* < 0.05)

Overexpression of *CsTIR1* and *CsAFB2* resulted in reduced seed size (Fig. 5a) and fewer seeds in each fruit (Fig. 5b). Further investigations of germination properties showed that the seed germination potential was significantly reduced in transgenic lines (Fig. 5c). To assess the molecular mechanisms underlying these alterations, the expression of *AP-like* (determiner for seed size in *Arabidopsis* [24]), *STK* (a maternal role in fertilization and seed development and related to seed number [25]), *GIGANTEA* and *ELIP* (involved in the seed germination process [26]) were investigated. Expression analysis revealed that *SlAP*-like, *SlSTK* and *SlGIGANTEA* were downregulated in the transgenic lines, while *SlELIP* was upregulated (Fig. 5d).

**Fig. 6:**
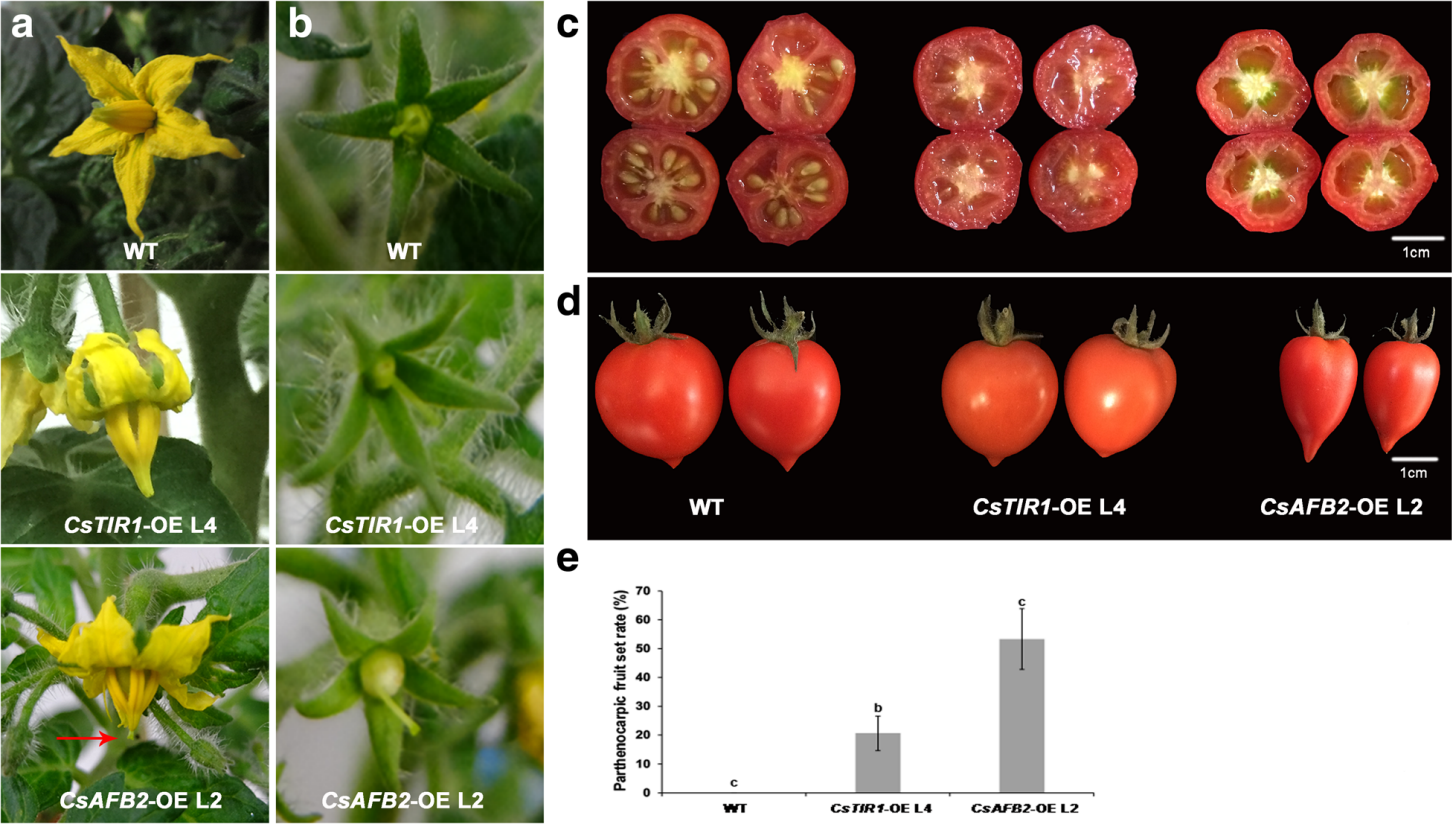
Fruit set and parthenocarpy in transgenic plants. **a** Flowers at anthesis day in wild-type and transgenic plants. Both *CsTIR1*-OE L4 and *CsAFB2*-OE L2 exhibited ovary expansion prior to anthesis, and splited staminal cone. The exposed stigma of *CsAFB2*-OE L2 is labeled with red arrow. **b** Ovaries of transgenic lines were successfully set fruit and expansion after emasculation. **c** Wild-type seeded fruit and transgenic parthenocarpic fruit. Scale bar = 1 cm. **d** Altered fruit shape was observed in the *CsAFB2*-OE L2. Scale bar = 1 cm. **e** Comparison of parthenocarpic fruit set rate among wild-type, *CsTIR1*-OE L4, and *CsAFB2*-OE L2 plants. Data represent mean ± SD of three biological replicates with more than twenty fruits for each replicate. Different letters above bars indicate significant differences among different genotypes (Student’s *t*-test, *P* < 0.05)

Although transcription analysis showed that *CsTIR1* and *CsAFB2* were downregulated during both the pollination and parthenocarpic fruit set processes, emasculation experiments suggested that overexpression of *CsTIR1* or *CsAFB2* induced facultative parthenocarpy in tomato fruit (Fig. 6a, b; Table 1). Seedless fruit phenotypes were also observed in both the *CsTIR1-*OE and *CsAFB2-*OE lines (Fig. 6c). Ovary expansion prior to anthesis may be the cause of seedlessness fruit phenotype (Fig. 6b). The *CsAFB2-OE* lines exhibited more obvious negative effects on seed set than the *CsTIR1-OE* lines because of the exposed stigma phenotype (Fig. 6a, e). Moreover, elongated fruit shape was induced by *CsAFB2* (Fig. 6d; Table 1). More detailed information about the phenotypes of *CsTIR1-*OE and *CsAFB2-*OE transgenic tomato plants is shown in Table 2.

**Table 2:** Phenotypes of wild-type (WT), *CsTIR1*-overxpressing (*CsTIR1*-OE) and *CsAFB*2-overexpressing (*CsAFB2*-OE) transgenic tomato plants

parameter	WT	*CsTIR1*-OE L1	*CsTIR1*-OE L2	*CsTIR1*-OE L3	*CsTIR1*-OE L4	*CsAFB2*-OE L1	*CsAFB2*-OE L2
Plant height (one month old, cm)	8.8 ± 0.2a	4.2 ± 0.3f	4.6 ± 0.2e	5.4 ± 0.1d	4.2 ± 0.2f	6.1 ± 0.3c	6.5 ± 0.1b
Plant height (three month old, cm)	20.5 ± 1.1a	9.4 ± 1.1c	9.9 ± 0.6c	8.6 ± 0.9c	8.2 ± 1.1c	13.6 ± 1.2b	12.3 ± 1.2b
Length of seed (mm)	4.4 ± 0.4a	3.0 ± 0.2b	3.1 ± 0.4b	3.2 ± 0.2b	3.2 ± 0.4b	3.3 ± 0.3b	3.3 ± 0.2b
Width of seed (mm)	2.8 ± 0.3a	1.5 ± 0.2b	1.6 ± 0.2b	1.5 ± 0.2b	1.5 ± 0.2b	1.5 ± 0.2b	1.6 ± 0.2b
Seeds per fruit (n)	16.5 ± 1.3a	13.1 ± 1.4b	13 ± 1.3b	12.9 ± 1.4b	13.2 ± 1.5b	3.5 ± 1.2c	3.4 ± 1.1c
Seeds germination rate (%)	98.7 ± 2.3%a	72 ± 6.9%b	70.6 ± 2.3%b	72 ± 6.9%b	69.3 ± 2.3%b	25.3 ± 4.6%c	21.3 ± 6.1%c
Parthenocarpic fruit set rate (%)	0c	20.8 ± 6.7%b	19.7 ± 5.9b	19.6 ± 5.5%b	20.6 ± 5.9%b	51.3 ± 11.0%a	53.3 ± 10.5%a
Horizontal diameters of fruit (cm)	2.1 ± 0.2a	2.0 ± 0.2b	1.8 ± 0.2b	1.9 ± 0.1b	1.9 ± 0.2b	1.4 ± 0.1c	1.4 ± 0.1c
Vertical diameters of fruit (cm)	3.3 ± 0.1a	2.8 ± 0.1c	2.9 ± 0.1bc	2.9 ± 0.1bc	2.9 ± 0.1bc	3.0 ± 0.1b	3.0 ± 0.1b

Values are means of 5–10 plants, ±SE. The statistical significance of mean differences was analyzed using Student’s *t*-test, *P* < 0.05

### Investigation of post-transcriptional regulations of *CsTIR1/AFB2* via miR393

**Fig. 7:**
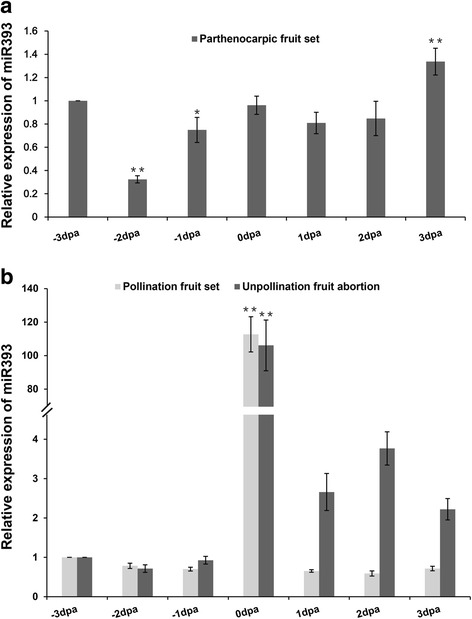
Expression analysis of miR393 during early stages of cucumber fruit development. **a** Relative expression of miR393 during the early stages of parthenocarpic fruit set development (−3 dpa to 3 dpa). dpa: days post-anthesis. **b** Relative expression of miR393 during the early stages of pollinated fruit set formation and unpollinated fruit abortion (−3 dpa to 3 dpa). Expression of miR393 in −3 dpa fruit was normalized to 1. Data represent mean ± SD of three biological replicates. (Student’s *t*-test; *P < 0.05; **P < 0.01)

Previous studies have demonstrated that miR393 plays a role in the regulation of TIR1/AFB expression [12, 27]. Despite the roles of miR393 and its target genes in multiple biological processes, much remains to be clarified about the function of miR393 and the miR393/TIR1 homologs module in regulating cucumber fruit set and development. MiR393 expression levels were analyzed in different early fruit developmental stages (Fig. 7). In parthenocarpic fruit, miR393 expression remained relatively stable, especially from 0 to 3 dpa (Fig. 7a), while in non-parthenocarpic fruit, miR393 expression was relatively low before anthesis but increased dramatically at the point of anthesis (0dpa). When the ovaries were pollinated manually (pollination induced fruit set), miR393 expression decreased rapidly to the low level detected in the immature ovaries (−3, −2, −1dpa). Although miR393 expression was also downregulated in abortive fruit (bagging treatment), it was 2–4-fold higher relative to that in the pollinated fruit (Fig. 7b).

**Fig. 8:**
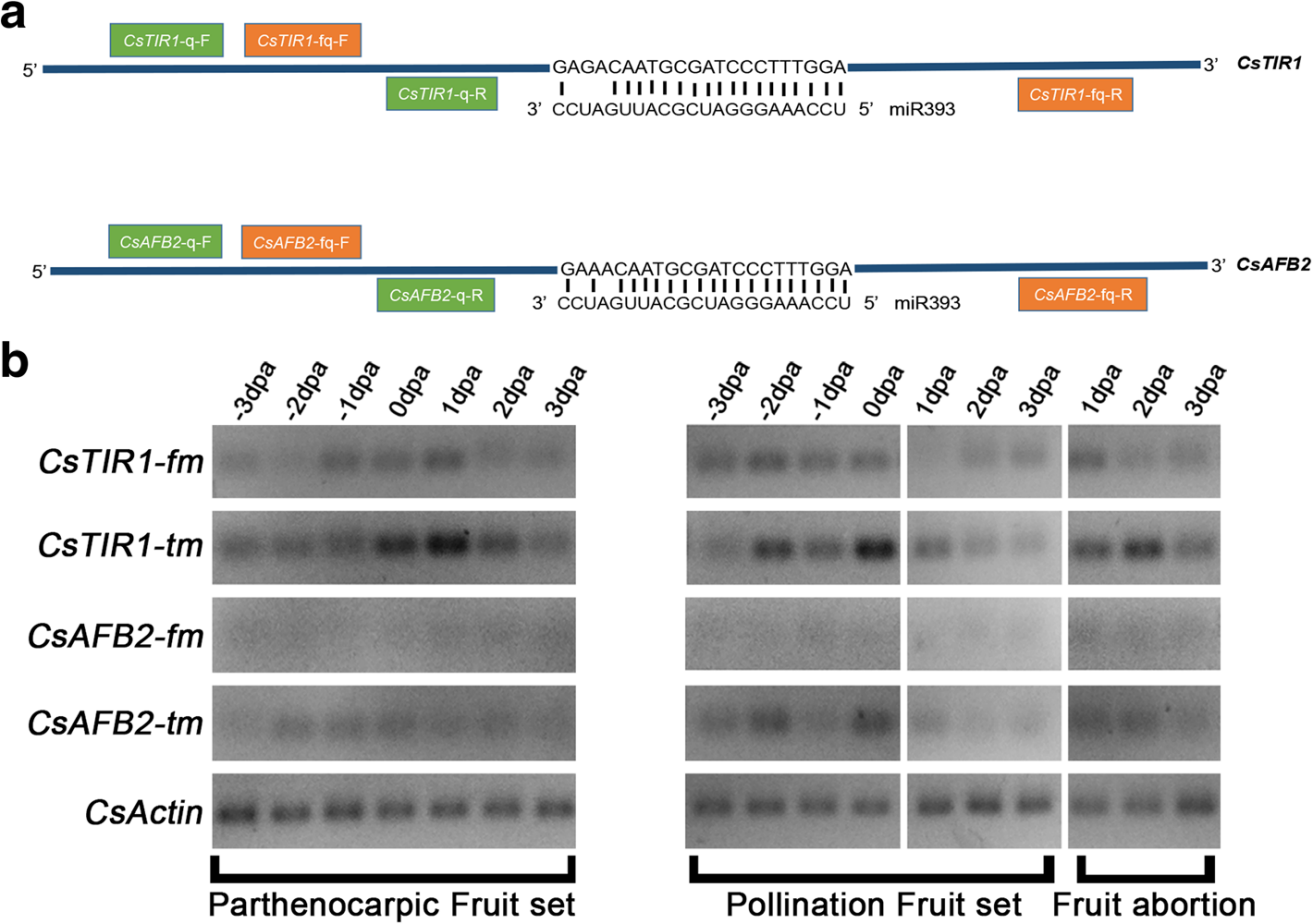
Expression analysis of full-length transcripts and total transcripts of *CsTIR1* and *CsAFB2* during the cucumber parthenocarpic fruit set, pollination fruit set and fruit abortion processes. **a** Schematic diagram of specific primer location. *CsTIR1* has three nucleotide mismatches in the miR393 recognition site, while *CsAFB2* contains two mismatches in the miR393 recognition site. The orange pair of primers separated on both sides of the miR393-cleavage site were used to detect the full-length transcripts of *CsTIR1* and *CsAFB2*. The green pair of primers distributed on one side of the miR393-cleavage site were used for detection of total transcripts of *CsTIR1* and *CsAFB2*. **b** Semi-quantitative RT-PCR analysis of full-length transcript and total transcript accumulation of *CsTIR1* and *CsAFB2* during the early stages of the cucumber parthenocarpic fruit set, pollination fruit set and fruit abortion processes (−3 dpa to 3 dpa). dpa: days post-anthesis. fm: full-length mRNA (complete fragments); tm: total mRNA (complete fragments and alternative splicing fragments)

In cucumber, *CsTIR1* has three nucleotide mismatches in the miR393 recognition site, while *CsAFB2* contains two mismatches, which are thought to cause different effects on miR393-directed mRNA cleavage (Fig. 8a). This suggests that *CsTIR1* and *CsAFB2* are regulated by miR393 in a post-transcriptional manner. To investigate miR393-mediated post-transcriptional regulations of CsTIR1/AFB2 during the fruit set process, four pairs of specific primers were designed for the detection of full-length transcripts (complete fragments) and total mRNA (complete fragments and alternative splicing fragments) of *CsTIR1* and *CsAFB2* (Fig. 8a; Additional file 3).

Both the total and full-length mRNA transcripts of *CsAFB2* were much less abundant than those of *CsTIR1*, and the full-length mRNA of *CsAFB2* was barely detected during these fruit developmental processes. The total transcripts of *CsTIR1* and *CsAFB2* reached the highest expression levels at 0dpa, while only low levels of the full-length transcripts of these genes were detected (Fig. 8b). In accordance with this, qRT-PCR analysis showed that miR393 expression peaked at anthesis (Fig. 7b), indicating that miR393-mediated cleavage of *CsTIR1* and *CsAFB2* was active during flowering (0dpa). Despite the relatively low miR393 expression levels, some full-length mRNA was detected in both pollinated and parthenocarpic fruit set (Figs. 7 and 8b). Interestingly, although the miR393 levels in abortive fruit were relatively higher than those of set fruit (Fig. 7b), there was almost no degradation of full-length mRNA of *CsTIR1* in abortive fruit (Fig. 8b).

## Discussion

As a reference species for *Cucurbitaceae* crops, cucumber exhibits various traits that may be regulated by various auxin gene networks. However, few auxin signaling transduction components of *cucumber* have been studied. To investigate the exact molecular mechanisms underlying the functions of cucumber auxin receptors in plant growth and fruit development, both *CsTIR1* and *CsAFB2* genes were isolated and the physiological and molecular consequences of their overexpression in tomato plants were evaluated.

In *Arabidopsis*, six auxin receptors have been identified, comprising *AtTIR1* and its closest paralogs *AFB1*- *AFB5* [10]. In tomato plants, three F-box receptor members have been identified [16], while three TIR1-like auxin receptors were found in plum [19] and there are six *TIR1/AFBs* homologs in the rice genome [17]. In *Populus*, eight *TIR1* homologous genes (*PtrFBL*s) were identified [28]. However, only two auxin receptors belonging to the F-box *TIR1*-like gene family have been identified in cucumber [29, 30]. Cucumber has a small genome with few tandem duplications [31]. Previous studies have shown that evolutionary momentum is basically facilitated by genome duplication events, which are thought to occur frequently during the evolution of organism [32, 33]. Therefore, it is tempting to speculate that the small number of TIR1 homologs in cucumber is probably due to the absence of recent whole-genome duplications.

*TIR1/AFBs* show auxin-insensitive transcriptional characteristics in *Arabidopsis* and *Populus* [12, 28]. Similarly, limited responses of *CsTIR1* to NAA treatment were also detected in cucumber, although *CsAFB2* showed sensitive responses to various exogenous phytohormones treatments (Fig. 3c, d). As intranuclear auxin receptors, TIR1/AFB proteins generally perform their functions within the nucleus [1, 16, 34]. However, subcellular localization analysis showed that cucumber CsTIR1 proteins were present not only in the nucleus but also on the cell membrane (Fig. 2b). Thus, the potential function of CsTIR1 as a membrane receptor deserves further investigation.

A series of studies suggested that the TIR1 protein may have a parallel function in leaf architecture and fruit development. Overexpression of *SlTIR1* results in pleiotropic phenotypes in tomato plants, including parthenocarpic fruit formation and leaf morphology [16]. El-Sharkawy demonstrated the critical role of *PslTIR1* as a positive regulator of auxin signaling in coordinating the development of leaves and fruit [19]. Furthermore, knockdown of either *OsTIR1* or *OsAFB2* altered the flag leaf inclination angle [17]. In this study, the highest transcription levels of *CsTIR1* and *CsAFB2* were in leaves and female flowers (Fig. 3a, b). Overexpression of the cucumber TIR1-like proteins induced curved growth of leaves and abnormal fruit expansion (Fig. 4a, b and 6a, b). Previous studies showed that trichome development is also regulated by TIR1 proteins [16]; however, no obvious alterations in trichome numbers and morphology were observed in the *CsTIR1*-OE and *CsAFB2*-OE transgenic lines. In addition, both the *CsTIR1*-OE and *CsAFB2*-OE lines exhibited decreased seed germination potential (Fig. 5c). Down-regulation of *SlGIGANTEA* in the transgenic lines is consistent with its positive role in the promotion of germination under continuous R in tomato seeds revealed in a previous study [35]. Furthermore, *SlELIP*, which has been reported to be significantly increased by inhibition of seed germination [26], was found to be upregulated in the transgenic lines. Thus, these observations indicate auxin receptor overexpression in tomato plants alters the expression of genes involved in seed germination process. Considering that silence of *AtTIR1* could also repress seed germination activity [1], therefore, it can be speculated that transcriptional balance of *TIR1* family genes is essential for seed germination.

Independent evidence suggests that TIR1/AFB proteins function as redundant auxin receptors, collectively mediating auxin-regulated responses throughout plant growth and development. Different phenotypes induced by overexpression of *CsTIR1* and *CsAFB2* were revealed in this study. *CsTIR1* and *CsAFB2* were found to have opposing regulatory functions in controlling the number of stomata, with *CsTIR1* overexpression inhibiting stomata formation*,* while stomata numbers were significantly increase by *CsAFB2* overexpression (Fig. 4c). Interestingly, *CsTIR1*-OE and *CsAFB2*-OE overexpression resulted in similarly opposing patterns of *SlSPCH* and *SlMUTE* expression (Fig. 4d), suggesting that overexpression of *CsTIR1* and *CsAFB2* has different effects on expression of stomata differentiation-related genes, thereby causing the opposite phenotypes in terms of stomata number. Furthermore, *CsAFB2*-OE lines exhibited higher parthenocarpic fruit set rates and altered fruit shape compared with the *CsTIR1*-OE lines. Studies have indicated that co-receptor complexes formed by different combinations of TIR1/AFB and the Aux/IAA proteins have a wide range of auxin-binding affinities [12, 28, 36]. We speculate that this accounts for the differences in auxin-related phenotypes, including alterations in leaf architecture and fruit development that are induced by *CsTIR1* and *CsAFB2* overexpression.

The roles of the miR393/TIR1 homolog module in regulating root growth, leaf inclination, tillering, disease resistance and salt tolerance have been well characterized. In *Arabidopsis*, regulation of auxin response by miR393-targeted TIR1 is involved in normal development [37], and the nitrate-responsive miR393/AFB3 regulatory module controls the root system architecture [36]*.* In addition, the miRNA393⁄TIR1 homolog module influences flag leaf inclination, crown root initiation, seminal root development and tillering in rice [17, 38]. Moreover, miR393-guided cleavage of TIR1, AFB2, and AFB3 transcripts enhances innate immunity in response to bacterial and fungal infection in leaves [27, 39]. Downregulation of *OsTIR1* and *OsAFB2* via *OsmiR393* led to reduced tolerance to salt and drought in rice [40]. In contrast, overexpression of a miR393-resistant form of mTIR1 enhanced salt tolerance in *Arabidopsis* [41]. However, less is known about the role of the miR393/TIR1 homolog module in the stages of fruit development. In this study, we also explored the mechanism by which the miR393/TIR1 homolog module regulates cucumber fruit development. We found that miR393regulates the degradation of *CsAFB2* in all types of fruit development, with total degradation of full-length transcripts of *CsAFB2* detected regardless of the process of parthenocarpic or pollinated fruit development or fruit abortion. Although miR393 expression levels were extremely high at anthesis, some full-length *CsTIR1* remained undegraded, probably due to the enrichment of *CsTIR1* transcripts or the incomplete cleavage of the miR393 target, *CsTIR1*. More interestingly, cleavage of *CsTIR1* was complete in 1dpa pollinated fruit, while there was no cleavage in 1dpa abortive fruit, in which even the miR393 level was much higher than that in the pollinated fruit (Fig. 8b). These findings provide an indication that the divergent functions of *CsTIR1* and *CsAFB2* are induced by the differences in cleavage regulation by miR393; however, the differences in miR393-mediated regulation of *CsTIR1* and *CsAFB2* cleavage remain to be verified through in vivo.

## Conclusion

This study was focused on the identification of distinct functions of cucumber TIR1-like genes. We identified and cloned both members of this gene family in cucumber. We demonstrated the differences in subcellular localizations of *CsTIR1* and *CsAFB2* proteins and analysis of *CsTIR1* and *CsAFB2* expression indicated their involvement in leaf morphogenesis and the fruit set process in cucumber. The roles of *CsTIR1* and *CsAFB2* in these processes were further revealed by the analysis of overexpressing lines. Moreover, qPCR and semi-qPCR results support the hypothesis that the miR393/TIR1 module participates in the cucumber fruit set process. These findings further clarify the functions of cucumber auxin receptors and provide new insights into the role of cucumber TIR1 homologs and miR393 in regulating fruit/seed set development and leaf morphogenesis.

## Methods

### Plant materials and growth conditions

Two cucumber breeding lines, parthenocarpic line ‘EC1’ and non-parthenocarpic line ‘8419 s-1’, were grown in greenhouses during the natural growing season (12 h photoperiod, 29/17 °C average day/night temperature, 85% humidity, 800 μmolm^−2^ s^−1^) at Nanjing Agricultural University (China). Both ‘EC1’ and ‘8419 s-1’ were breeding materials of the Nanjing Agricultural University. Tissues (root, stem, leaf, female flower, male flower and ovary) were collected from 9-week-old ‘8419 s-1’ plants. For hormone treatment analysis, the leaves of ‘8419 s-1’ plants (*n* = 30 per group) were treated with GA_3_ (10 μM), 6-BA (10 μM) and different concentrations of NAA (5, 10, and 50 μM) and collected at 6 h after treatments. Ovaries were treated by pollination and with NAA (500 μM), GA3 (3000 μM), CPPU (400 μM), or BRs (0.2 μM) at anthesis and collected at 6 h after treatments. Thirty ‘EC1’ plants and thirty ‘8419 s-1’ plants were used for fruit set and development analysis. Female flowers at the 12–15th node of the main stem were isolated to prevent pollen contamination on the day before anthesis, followed by treatment as above with bagging and pollination (‘EC1’ bagging represents the parthenocarpic fruit set process, ‘8419 s-1’ bagging represents unpollination fruit abortion process, ‘8419 s-1’ pollination represents pollinated fruit set process). The ovaries of the treated female flowers were harvested at −3, − 2, −1, 0, 1, 2, 3 days post-anthesis (dpa). Non-treated leaves and fruit were used as controls. All samples were frozen in liquid nitrogen, and stored at −80 °C prior to analysis. Sampling was performed on three independent occasions.

Tomato plants (*Solanum lycopersicum,* cv MicroTom) were used in this study to generate *CsTIR1* and *CsAFB2* overexpression lines. The seeds of MicroTom were kindly provided by Professor Zhengguo Li (Genetic Engineering Research Center, Chongqing University, China). All tomato plants (10 plants per each line) were grown in an illuminated incubator under controlled conditions set as follows: 14 h day/10 h night cycle; 25/20 °C day/night temperature; 80% relative humidity and 250μmolm^−2^ s^−1^ light intensity.

### Bioinformatics analysis of *CsTIR1* and *CsAFB2*

The cucumber genome database (http://cucumber.genomics.org.cn/page/cucumber/index.jsp) was searched for homologs of TIR1-like auxin receptors in cucumber using the *AtTIR1* (AT3G62980.1) sequence. Blast searches revealed two cucumber genes (*Csa001802* and *Csa015043*) with the highest similarity to *AtTIR1*, and two primer pairs were designed to amplify the full-length cDNA sequences of these two genes using cDNA of cucumber fruits as a templates (Additional file 4). The PCR products were directly sequenced (Invitrogen) and sequence data were submitted to GenBank (accession number: GX901282 and GX901283). The MEGA program (version 5) was used for phylogenetic analysis with known TIR1-like auxin receptors based on homology. Structural domains were annotated using Smart (http://smart.embl-heidelberg.de/) and illustrated using prosite (http://prosite.expasy.org/mydomains/) with default parameters. GenBank accession numbers for the sequences analysis are listed in Additional file 5.

### Subcellular localization of CsTIR1-GFP and CsAFB2-GFP fusion protein

The open reading frames of *CsTIR1* and *CsAFB2* were amplified and cloned into the pGreen0029 vector without the stop codon using specific primers (Additional file 4). Recombinant plasmids (pGreen 0029- *CsTIR1-GFP* and pGreen 0029-*CsAFB2-GFP*) and a control plasmid with *GFP* alone were introduced into onion epidermal cells (obtained from Lab of Cucurbit Genetics and Germplasm Enhancement, Nanjing Agricultural University) using 1.0 μm of gold microcarriers delivered via a pneumatic particle gun (Bio-Rad, PDS-1000/He, USA) with the following bombardment conditions: vacuum, 635 mmHg; helium pressure, 1100 psi; target distance, 6 cm. After bombardment, onion epidermis cells were cultured on MS (Murashige and Skoog) medium for 14 h at 25 °C in darkness. The transformed cells were visualized using a confocal laser scanning microscope (Zeiss, LSM780, Germany).

### Generation of transgenic tomato plant lines

The full-length sequences of *CsTIR1* and *CsAFB2* were amplified using gene specific primers (Additional file 4). Fragments of *CsTIR1* and *CsAFB2* were cloned into plp100 binary vector under the transcriptional control of the *35S* promoter. The constructs were then introduced into WT tomato plants (*Solanum lycopersicum* cv MicroTom) by *Agrobacterium*-mediated transformation [16]. The *Agrobacterium* (C58) was kindly provided by Professor Zhengguo Li (Genetic Engineering Research Center, Chongqing University, China). Transformed lines were selected on kanamycin (50 mg L^−1^) and further analyzed by qRT-PCR to confirm the presence of T-DNA inserts in the transgenic lines. For each construct, more than six independent lines with consistent phenotypes were obtained. Homozygous lines from the T2 generation were used for experiments.

### Electron microscopy

Segments of leaves were collected from WT and transgenic plants after 6 weeks of growth on soil. Three replicates of each sample were mounted on aluminum stubs, sputtered with gold palladium for 30s, and examined under an S-3000 N scanning electron microscope (Hitachi, Japan).

### Evaluation of parthenocarpy and seed germination assays

To evaluate parthenocarpic fruit set rates of transgenic lines and WT plants, flower buds (5 plants per line) were emasculated 2 d before anthesis to prevent self-pollination. To guarantee equivalent growth conditions for parthenocarpic fruit, only seven flowers were kept per plant. The parthenocarpic fruit set rate was represented by the percentage of emasculated flowers that developed into fruit.

For seed germination assays, seeds were obtained and counted from WT and transgenic tomato lines (5 plants per line). The seeds were placed in petri dishes containing a layer of wet 3 mm filter paper (Whatman, China) and incubated in the dark at 25 °C. The number of germinated seeds was counted after 3 days. The emergence of a radicle was considered as a germination event for the calculation of the percent germination. The experiment was replicated on three independent occasions with 25 seeds per replicate. All phenotypic data was listed in Additional file 6.

### Gene expression analysis

All primers used for qRT-PCR and semi-quantitative RT-PCR are listed in Additional file 3. Total RNA was isolated using TRIzol reagent (Invitrogen, USA) and treated with DNase I (Fermentas, UK) according to the to manufacturer’s instructions. First-strand cDNA was synthesized using the PrimeScript™ RT-PCR Kit (TaKaRa, Japan). Real-time quantitative RT-PCR was then carried out using the SYBR® Premix Ex Taq™ Kit (TaKaRa, Japan) in a CFX96 multicolor real-time PCR detection system (Bio-Rad, USA). *Sl-actin* and *Cs-actin* were used as the internal control genes. For qRT-PCR analysis of *Cs-miR393*, U6 was used as internal control gene and the first-strand cDNA was synthesized using Mir-X miRNA First-Strand Synthesis Kit (TaKaRa, Japan). The qRT-PCR was carried out using the SYBR® Premix Ex Taq™ II Kit (TaKaRa, Japan). Quantification of mRNA and miRNA levels was based on the comparative cycle threshold (CT) method and calculated as 2^-ΔΔCT^. Analysis was conducted on the data from three independent reactions (technical replicates) using samples from three biological replicates. All CT values were listed in Additional file 7.

## Additional files

Additional file 1: Fig. S1. Comparison of the predicted amino acid sequences of cucumber CsTIR1, CsAFB2 and AtTIR1, VvAFB2. (PDF 386 kb)

Additional file 2: Fig. S2. Quantitative PCR assession of positive transgenic tomato lines and detection of *SlTIR1* expression in transgenic tomato lines. (PDF 132 kb)

Additional file 3: Table S1. The oligonucleotide primer for qRT-PCR and semi-quantitative RT-PCR. (DOCX 14 kb)

Additional file 4: Table S2. List of primers used in construct preparation. (DOCX 15 kb)

Additional file 5: Table S3. Sequences from TIR-like F-box proteins of Embryophyte species used to generate the phylogenetic tree. (DOCX 15 kb)

Additional file 6: Table S4. Phenotypic data of Wild-type, CsTIR1-OE lines and CsAFB2-OE lines. (XLSX 10 kb)

Additional file 7: Table S5. CT values of RT-PCR experiments conducted in this study. (XLSX 23 kb)

## Abbreviations

6-BA: 6-benzylamino-purine
BRs: Brassinosteroids
CPPU: N-(2-chloro-4-pyridyl)-N0-phenylurea, a diphenylurea-derived cytokinin
dpa: days post anthesis
GA3: Gibberellic acid
GFP: Green Fluorescent Protein
NAA: Naphthyl acetic acid
qRT-PCR: Quantitative real-time PCR
