# Supplementary material for: New insights into the roles of cucumber TIR1 homologs and miR393 in regulating fruit/seed set development and leaf morphogenesis
Source: BMC Plant Biol. 2017 Jul 26;17:130. doi: 10.1186/s12870-017-1075-6 (PMC5530481; doi:10.1186/s12870-017-1075-6)
Supplement: Supplementary file 1 — Comparison of the predicted amino acid sequences of cucumber CsTIR1, CsAFB2 and AtTIR1, VvAFB2. (PDF 386 kb) [file 12870_2017_1075_MOESM1_ESM.pdf]

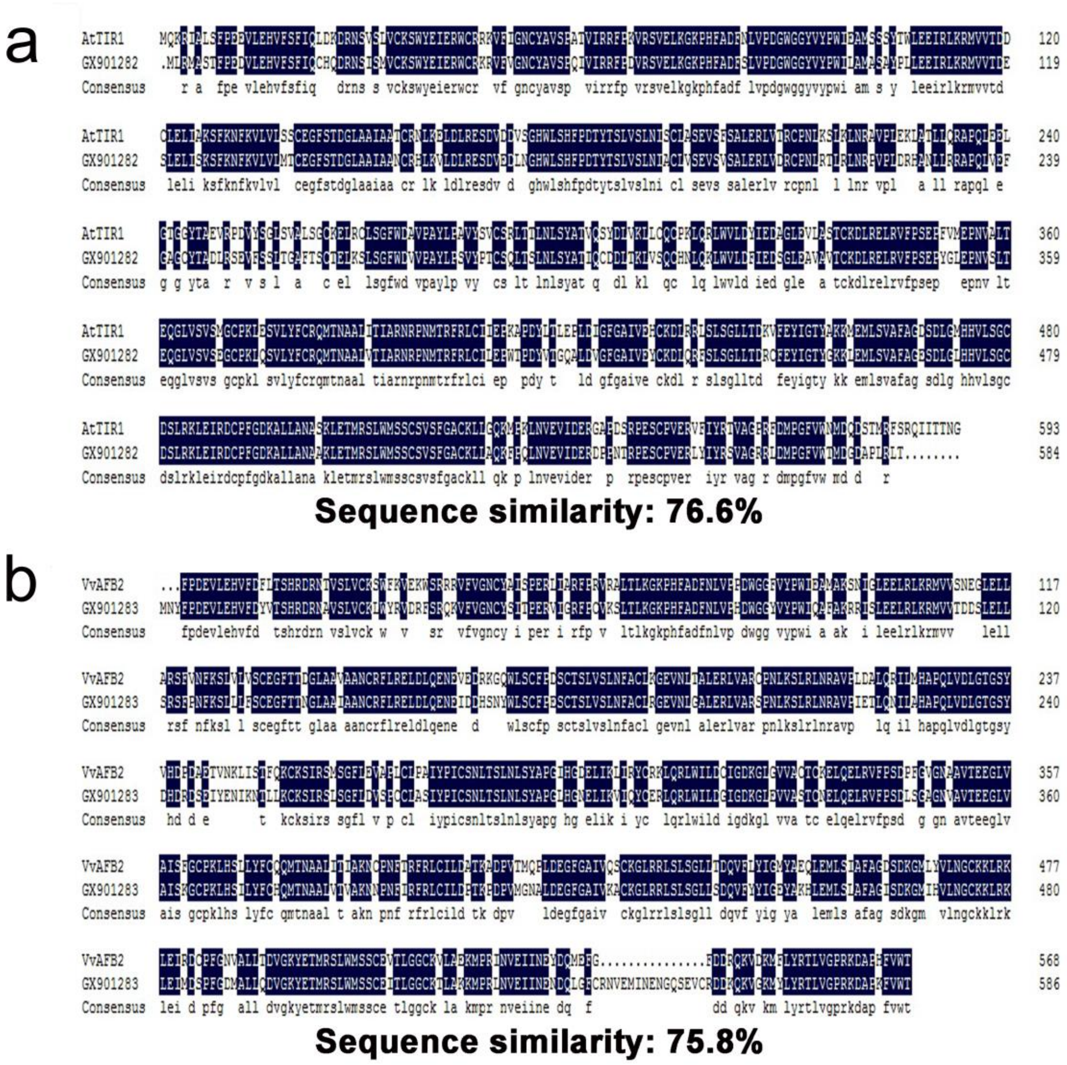

Additional file 1: Fig S1. Comparison of the predicted amino acid sequences of cucumber CsTIR1, CsAFB2 and AtTIR1, VvAFB2. **a** Alignment of the predicted amino acid sequences of GX901282 (CsTIR1) and AtTIR1. Their sequence similarity is 76.6%. **b** Alignment of the predicted amino acid sequences of GX901283 (CsAFB2) and VvAFB2. They have 75.8% sequence similarity. The numbers on the right indicate the positions of the amino acid residues. Identical amino acids are shaded in navy blue
