# Supplementary material for: New insights into the roles of cucumber TIR1 homologs and miR393 in regulating fruit/seed set development and leaf morphogenesis
Source: BMC Plant Biol. 2017 Jul 26;17:130. doi: 10.1186/s12870-017-1075-6 (PMC5530481; doi:10.1186/s12870-017-1075-6)
Supplement: Supplementary file 2 — Quantitative PCR assession of positive transgenic tomato lines and detection of SlTIR1 expression in transgenic tomato lines. (PDF 132 kb) [file 12870_2017_1075_MOESM2_ESM.pdf]

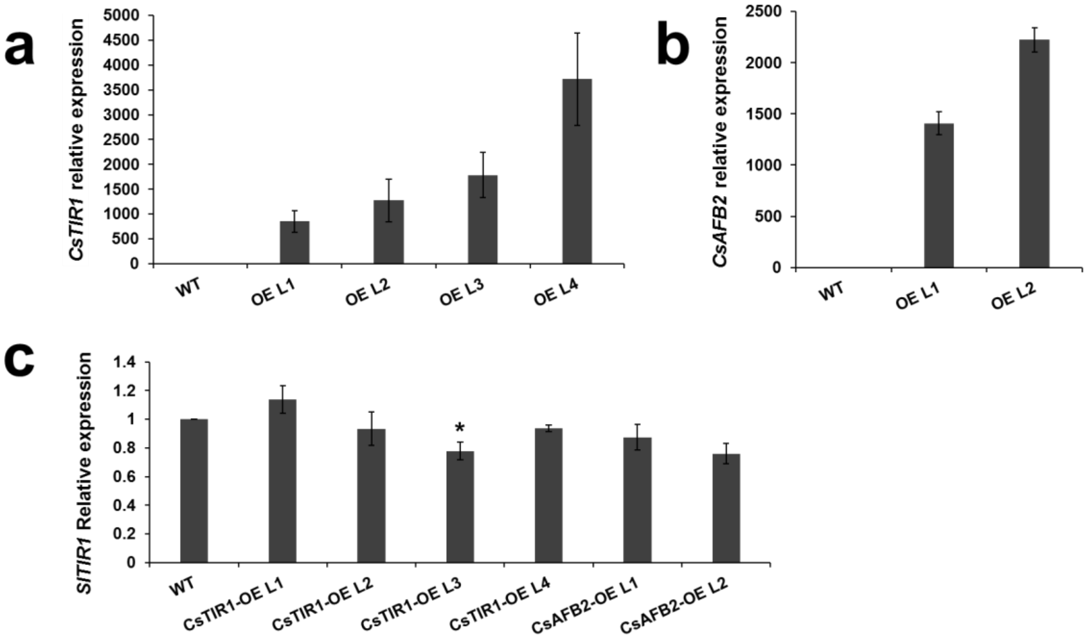

Additional file 2: Fig S2. Quantitative PCR assession of positive transgenic tomato lines and detection of *SlTIR1* expression in transgenic tomato lines. **a** Overexpressing of *CsTIR1* in transgenic tomato plants. Four *CsTIR1*-OE lines exhibited relative high amount of *CsTIR1* transcripts accumulation. **b** Overexpressing of *CsAFB2* in transgenic tomato plants. Two *CsAFB2*-OE lines exhibited relative high amount of *CsAFB2* transcripts accumulation. **c** Expression analysis of *SlTIR1* in both *CsTIR1*-OE and *CsAFB2*-OE lines. Error bars show the standard error between three biological replicates performed (n = 3). The expression data of WT were normalized to 1, respectively. The significant differences are calculated respect to expression inWT. *Asterisks* indicate significant differences (*t* test; *< 0.05)
