# Supplementary material for: New insights into the roles of cucumber TIR1 homologs and miR393 in regulating fruit/seed set development and leaf morphogenesis
Source: BMC Plant Biol. 2017 Jul 26;17:130. doi: 10.1186/s12870-017-1075-6 (PMC5530481; doi:10.1186/s12870-017-1075-6)
Supplement: Supplementary file 3 — The oligonucleotide primer for qRT-PCR and semi-quantitative RT-PCR. (DOCX 14 kb) [file 12870_2017_1075_MOESM3_ESM.docx]

**Additional file 3: Table S1.** The oligonucleotide primer for qRT-PCR and semi-quantitative RT-PCR

| **Application** | **Name** | **Oligonucleotide sequence** |
| --- | --- | --- |
| qRT-PCR | CsActin-F | 5'-F:TTCTGGTGATGGTGTGAGTC-3' |
|  | CsActin-R | 5'-GGCAGTGGTGGTGAACATG-3' |
|  | CsTIR1-q-F | 5'-GTGCCATTGTTGAGTATTGC-3' |
|  | CsTIR1-q-R | 5'-CTCCAGCTTGCGAAGACTAT-3' |
|  | CsAFB2-q-F | 5'-GTCCTGCGAGATTACTCTTG-3' |
|  | CsAFB2-q-R | 5'-AGACTTCTGACTGACCATTCT-3' |
|  | U6snRNA-F | 5'-GGGGACATCCGATAAAATT-3' |
|  | U6snRNA-R | 5'-TGTGCGTGTCATCCTTGC-3' |
|  | miR393 5' primer | 5'-TCCAAAGGGATCGCATTGATCC-3' |
|  | SlActin-F | 5'-TGTCCCTATTTACGAGGGTTATGC-3' |
|  | SlActin-R | 5'-CAGTTAAATCACGACCAGCAAGAT-3' |
|  | SlARF10-F | 5'-CTCGGTTCTTACTCTTCGGTC-3' |
|  | SlARF10-R | 5'-CCCTGTTTGGACTACTTGTG-3' |
|  | SlSPCH-F | 5'-CGACGATTCCGAATGCGATAT-3' |
|  | SlSPCH-R | 5'-TTCTTCTGACACCAAACCCGTAA-3' |
|  | SlMUTE-F | 5'-GTTCTTCAATCTCTTGAGGCGAAA-3' |
|  | SlMUTE-R | 5'-GGACTACTTTCAGGTGTAGGACTT-3' |
|  | SlSTK-F | 5'-CAACAACTTGTCCTGTGCCT-3' |
|  | SlSTK-R | 5'-TCCACAACAATGGTTGCAATA-3' |
|  | SlAP-like-F | 5'-ACGTTTAGGTGACGTGGTGA-3' |
|  | SlAP-like-R | 5'-TGCTCCGTTTCACTATTCCA-3' |
|  | SlGIGANTEA -F | 5'-GTCCAAGTGCCACACAGAGG-3' |
|  | SlGIGANTEA -R | 5'-GCAGAATCTGCAGCAATTCC-3' |
|  | SlELIP-F | 5'-TGTAGCAGCCATTGGTATGGAGC-3' |
|  | SlELIP-R | 5'-TCAGCAATGCACTTGACCCCAAG-3' |
|  | SlTIR1-F | 5'-AGGGGTCCTCCAGATACAAG-3' |
|  | SlTIR1-R | 5'-CGCTAATACCTGCCCATCTTT-3' |
| semi-quantitative RT-PCR | CsTIR1-fq-F | 5'-TGCAGGCGAGAGTGATTTAG-3' |
|  | CsTIR1-fq-R | 5'-AGCCTCTCCACAGGACAACT-3' |
|  | CsAFB2-fq-F | 5'-GTTTGGTGACATGGCACTTC-3' |
|  | CsAFB2-fq-R | 5'-ACGTTCAGCCTAGGCATCTT-3' |
