# Supplementary material for: New insights into the roles of cucumber TIR1 homologs and miR393 in regulating fruit/seed set development and leaf morphogenesis
Source: BMC Plant Biol. 2017 Jul 26;17:130. doi: 10.1186/s12870-017-1075-6 (PMC5530481; doi:10.1186/s12870-017-1075-6)
Supplement: Supplementary file 4 — List of primers used in construct preparation. (DOCX 15 kb) [file 12870_2017_1075_MOESM4_ESM.docx]

**Additional file 4: Table S2.** List of primers used in construct preparation

| **Construct** | **primer name** | **Forward** | **Reverse** |
| --- | --- | --- | --- |
| *CsTIR1* cDNA | *CsTIR1*-CDS | 5'-CAAACCGAAATTAGGGCAACA-3' | 5'-TCGATCCTCGGTTTGGTG-3' |
| *CsAFB2* cDNA | *CsAFB2-*CDS | 5'-TTGGATGCTGAGAAACGATG-3' | 5'-AAATTCAAAAGCCTTGATGGA-3' |
| *CsTIR1-GFP* | *CsTIR1*-fuse | 5'-TTTGTCGACCAAACCGAAATTAGGGCAACA-3' | 5'-TTTTCTAGAAGTAAGCCTTAGAGGAGCATCTC-3' |
| *CsAFB2-GFP* | *CsAFB2-*fuse | 5'-TTTAAGCTTTTGGATGCTGAGAAACGATG-3' | 5'-TTTGTCGACCAATGTCCATACAAACTTAGG-3' |
