# Supplementary material for: New insights into the roles of cucumber TIR1 homologs and miR393 in regulating fruit/seed set development and leaf morphogenesis
Source: BMC Plant Biol. 2017 Jul 26;17:130. doi: 10.1186/s12870-017-1075-6 (PMC5530481; doi:10.1186/s12870-017-1075-6)
Supplement: Supplementary file 5 — Sequences from TIR-like F-box proteins of Embryophyte species used to generate the phylogenetic tree. (DOCX 15 kb) [file 12870_2017_1075_MOESM5_ESM.docx]

**Additional file 5: Table S3.** Sequences from TIR-like F-box proteins of Embryophyte species used to generate the phylogenetic tree

| **Protein** | **Species** | **Protein length (aa)** | **Accession number** |
| --- | --- | --- | --- |
| CsTIR1 | *cucumis sativus.*L | 584 | JX901282 |
| CsAFB2 | *cucumis sativus.L* | 587 | JX901283 |
| AtTIR1 | *Arabidopsis thaliana* | 594 | NP_567135.1 |
| AtAFB2 | *Arabidopsis thaliana* | 575 | NP_566800.1 |
| AtAFB3 | *Arabidopsis thaliana* | 577 | NP_563915.1 |
| AtAFB4 | *Arabidopsis thaliana* | 623 | NP_567702.2 |
| AtAFB5 | *Arabidopsis thaliana* | 619 | NP_568718.1 |
| AtCOI1 | *Arabidopsis thaliana* | 592 | NP_565919.1 |
| BrTIR1 | *Brassica rapa* | 595 | XP_009116967.1 |
| BrAFB2 | *Brassica rapa* | 575 | XP_009129474.1 |
| CaTIR1 | *Cicer arietinum* | 583 | XP_004495993.1 |
| CaAFB2 | *Cicer arietinum* | 571 | XP_004493010.1 |
| CsoTIR1 | *Citrus sinensis* L. Osbeck | 585 | XP_006479877.1 |
| CsoAFB2 | *Citrus sinensis* L. Osbeck | 570 | KDO47371.1 |
| CsoCOI1 | *Citrus sinensis* L. Osbeck | 597 | KDO69096.1 |
| DlTIR1 | *Dimocarpus longan* | 586 | ACX31301.2 |
| FvTIR1 | *Fragaria vesca subsp. Vesca* | 584 | XP_004309692.1 |
| FvAFB2 | *Fragaria vesca subsp. Vesca* | 572 | XP_004304555.1 |
| GhTIR1 | *Gossypium hirsutum* | 586 | NP_001314527.1 |
| GmTIR1 | *Glycine max* | 585 | XP_003518950.1 |
| GmAFB2 | *Glycine max* | 572 | XP_003553972.1 |
| MaTIR1 | *Musa acuminata subsp. malaccensis* | 582 | XP_009393237.1 |
| MdTIR1 | *Malus domestica* | 587 | XP_008368919.1 |
| MdAFB2 | *Malus domestica* | 570 | XP_008379063.1 |
| NtTIR1 | *Nicotiana tabacum* | 581 | ACT53268.1 |
| OsTIR1 | *Oryza sativa* | 587 | XP_015639834.1 |
| OsAFB2 | *Oryza sativa* | 575 | XP_015635915.1 |
| PbAFB2 | *Pyrus x bretschneideri* | 572 | XP_009344196.1 |
| PsTIR1 | *Prunus salicina* | 584 | AIT18058.1 |
| PsAFB2 | *Prunus salicina* | 572 | AIT18061.1 |
| PsAFB5 | *Prunus salicina* | 632 | AIT18064.1 |
| RcTIR1 | *Ricinus communis* | 585 | XP_002520681.1 |
| RcCOI1 | *Ricinus communis* | 602 | XP_002530419.1 |
| SlTIR1 | *Solanum lycopersicum* | 581 | NP_001234673.1 |
| SlCOI1 | *Solanum lycopersicum* | 603 | NP_001234464.1 |
| VvTIR1 | *Vitis vinifera* | 576 | XP_002269127.1 |
| VvAFB2 | *Vitis vinifera* | 572 | XP_019080656.1 |
| VvCOI1 | *Vitis vinifera* | 598 | XP_002276145.2 |
| ZmTIR1 | *Zea mays* | 575 | XP_008669494.1 |
